# Supplementary material for: Voltage gating of mechanosensitive PIEZO channels
Source: Nat Commun. 2018 Mar 15;9:1096. doi: 10.1038/s41467-018-03502-7 (PMC5854696; doi:10.1038/s41467-018-03502-7)
Supplement: Supplementary file 1 — Supplementary Information [file 41467_2018_3502_MOESM1_ESM.pdf]

Voltage-gating of mechanosensitive PIEZO channels

Moroni *et al.*

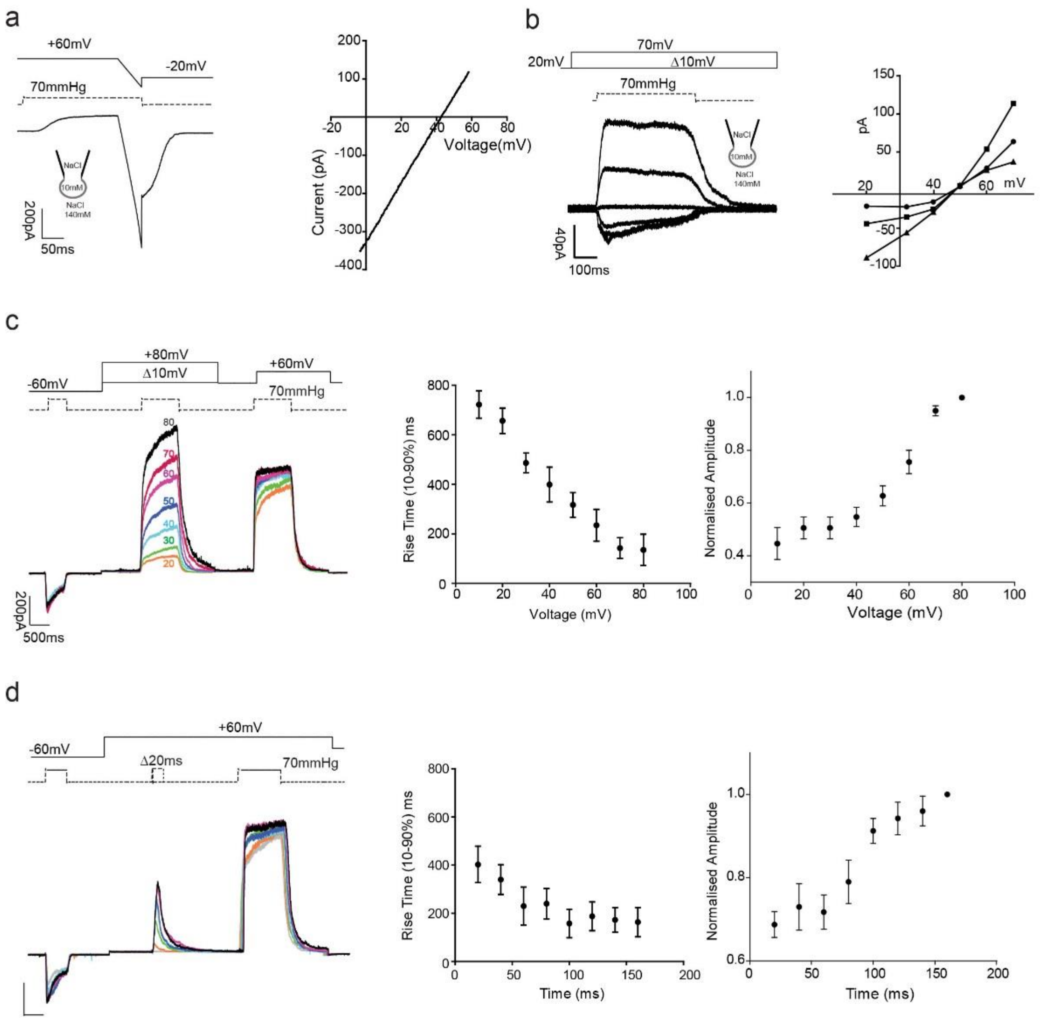

Supplementary Figure 1

Supplementary Figure 1. Recovery from Inactivation of PIEZO1 channels a) Left. Determination of reversal potential for PIEZO1 in presence of $[NaCl]_{in}$ (10mM)/$[NaCl]_{out}$ (140mM). Patches were held at +60mV and a saturating pressure step was applied for 100ms. Upon reaching steady state a voltage ramp (+60 to -40mV in 50ms) was applied. Right, example plot of the ramp current values plotted against voltage. The average E_{rev} value was +43 ± 1mV, 4 cells). b) I/V relationship of wt PIEZO1 with the same solutions as in a. Pressure steps were elicited at increasing voltages ranging from 20 to 70 mV in 10mV steps. E_{rev} : 45 ± 2mv, 3 cells

c) Paired pulses protocol of Fig. 2J was modified to measure the recovery from inactivation. An additional step was added between a first pressure step at -60mV and a pressure step at +60mV. During the second (test) pressure step the voltage was increased from 20mV to 80mV in successive sweeps with a 10mV increment. The amplitude and the rise time of the third step was then plotted (right) against the size of the test voltage step. Notice how the increase in the voltage of the second step is accompanied by an increase both in amplitude and rise time, thus underlying a faster recovery from inactivation with increasing voltage. d) The protocol in C is repeated. The voltage of the second step is kept constant at +60mV, while the duration is increased from 20ms to 160ms (20ms grey, 40ms orange, 60ms green, 80ms light blue, 100ms blue, 120ms magenta, 140ms red, 160ms black). Notice how steps as short as 40ms can recover up to 90% of the channels. For both c and d, amplitudes were measured averaging the amplitude values between 10 and 20 ms after the application of the third pressure step.

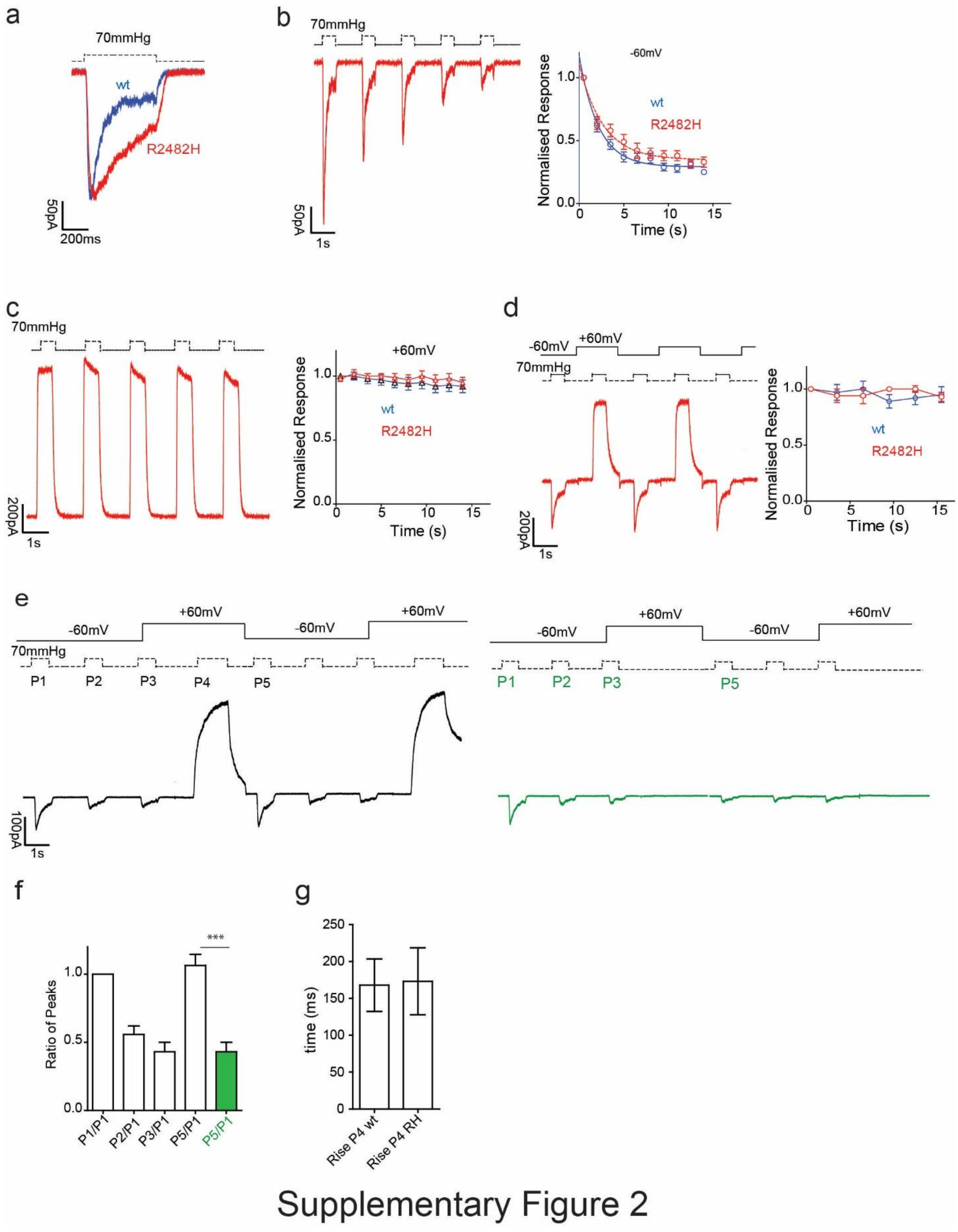

Supplementary Figure 2

Supplementary Figure 2. PIEZO1 R2482H desensitization and inactivation properties. (Related to Fig. 4)

a) R2482H (red trace) shows a slow kinetic of inactivation in comparison to the wild type (blue trace) as previously reported. b) Repetitive pressure stimulations desensitizes R2482H -60mV (b) but not at +60mV (c). As for the wild type at -60mV the fifth stimulus is much smaller than the first one and the current shows a decreased peak current and low peak/steady state current ratio. d) Alternating pressure pulses at -60mV and +60mV abolishes desensitization of PIEZO1 R2482H.

e) *Left*. 3 pressure pulses (P1, P2,P3) at -60mV were applied to patches expressing PIEZO1 R2482H to drive the channel into a desensitized state, followed by 1 positive pressure pulse (P4) at +60mV, as described in Fig 2. The sequence was repeated twice. As for the wild type outward permeation (P4) recovers PIEZO1 R2482H initial current (P1) and resets its time course for inactivation, as shown by the ratio between the current amplitude of P5/P1, shown in f). The stimulation sequence in e was repeated in absence of pressure to prevent channel opening and permeation when switching to a positive voltage (P4). The recovery from desensitization does not occur in absence of a permeation event. As observed for the wild type note the slow rise time of P4, most likely due to a slow transition out of the desensitized state. The rise time of P4 for wild type and R2482H channel are plotted in g. Values are not significantly different (Student's *t*-test $P<0.87$).

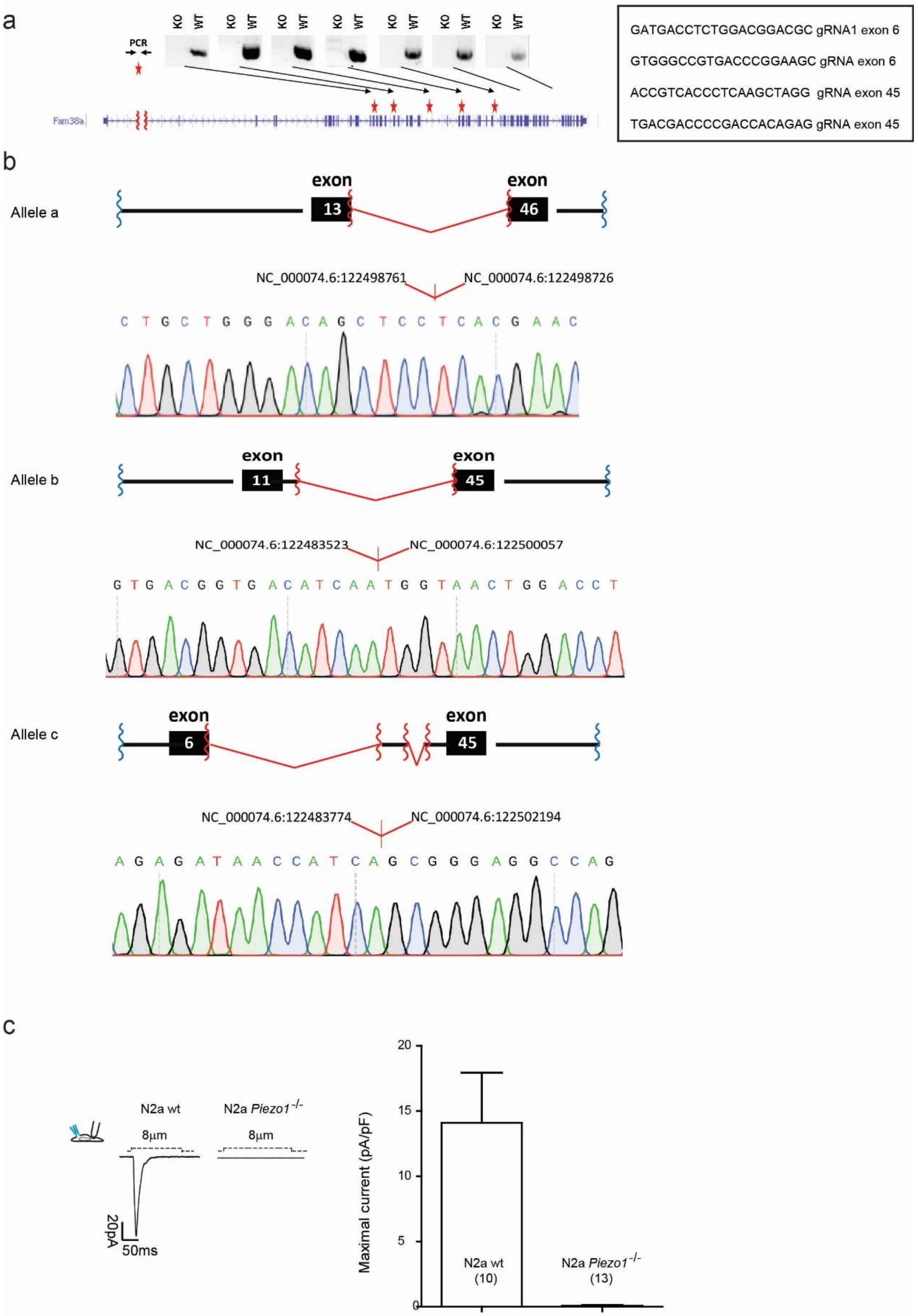

Supplementary Figure 3

Supplementary Figure 3. Generation of mouse N2a ***Piezo1 -/-*** **cell line (Related to Fig. 5 and 7)**

a) Left. Representation of the genomic locus of the mouse Piezo1 gene. Red stars indicated the loci where genotyping PCR were carried out to verify the absence of the genome in the KO clone. Right. gRNA oligos were cloned into the Cas9n plasmid (Addgene 48140) targeting exon 6 and exon 45. b) 3 copies of Piezo1 were detected in our N2a cells and for each allele 30 exons were deleted. N2a $^{Piezo1\ -/-}$ cells were sequenced over the junction points of the deleted alleles and the chromatograms of the sequencing reactions for each allele are reported. c) wt N2a and N2a $^{Piezo1\ -/-}$ cells were voltage clamped at -60 and tested for the presence of mechanically activated currents by means of soma indentation stimulation. Up to 8μm indentation stimuli produced high currents in N2a wild type cells, while they did not induce any current in N2a $^{Piezo1\ -/-}$ cells.

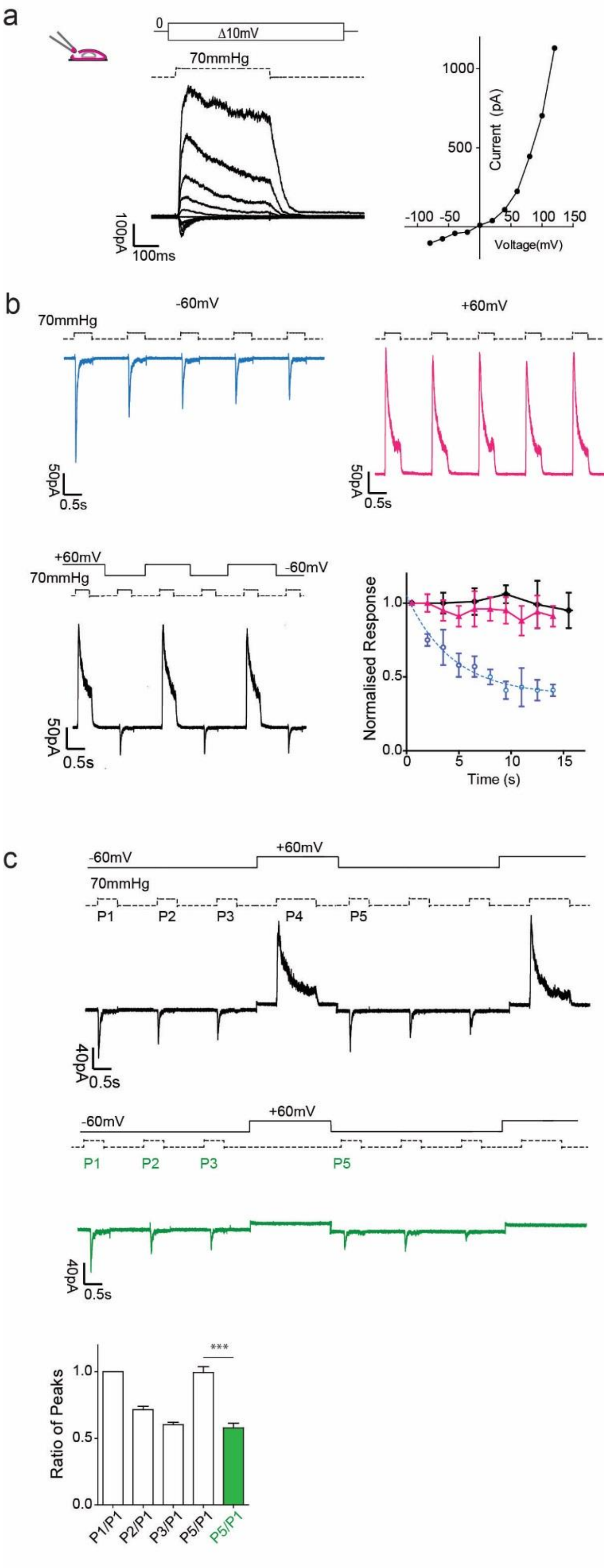

Supplementary Figure 4

Supplementary Figure 4. Biophysical properties of the PIEZO1-PIEZO2 chimera (Related to Fig. 5)

a) *Left*. Example traces of currents elicited at a constant saturating pressure (70mmHg) and at increasing voltages (in 20mV steps from -100mV to 100mV) in symmetrical Na^+ from excised (outside-out) patches overexpressing chimeric PIEZO1-PIEZO2 construct in N2a *Piezo1*$^{-/-}$ cells. *Right.* Peak currents are plotted against voltage to show an I/V relationship. Note the outwardly rectifying behavior. b) Consecutive pulses of saturating pressure at -60mV (blue traces), +60mV (magenta) and alternating -60/+60mV (black) were applied to outside-out patches expressing the chimeric PIEZO1-PIEZO2 construct. As for wild type mPIEZO1 inward currents elicited a fast onset of desensitization at negative voltages, lack of desensitization at positive or alternating voltages. c) As for mPIEZO1 outward permeation facilitated recovery from desensitization induced by repetitive inward currents. 3 pressure pulses (P1, P2,P3) at -60mV were applied to patches expressing the chimeric channel to drive the channel into an desensitized state, followed by 1 positive pressure pulses (P4) at +60mV. The sequence was repeated twice. Outward permeation (P4, black trace) allows the chimeric channel to recover from desensitization as shown by the ratio of P5/P1. Absence of outward permeation (P4, green trace) does not allow recovery from desensitization.

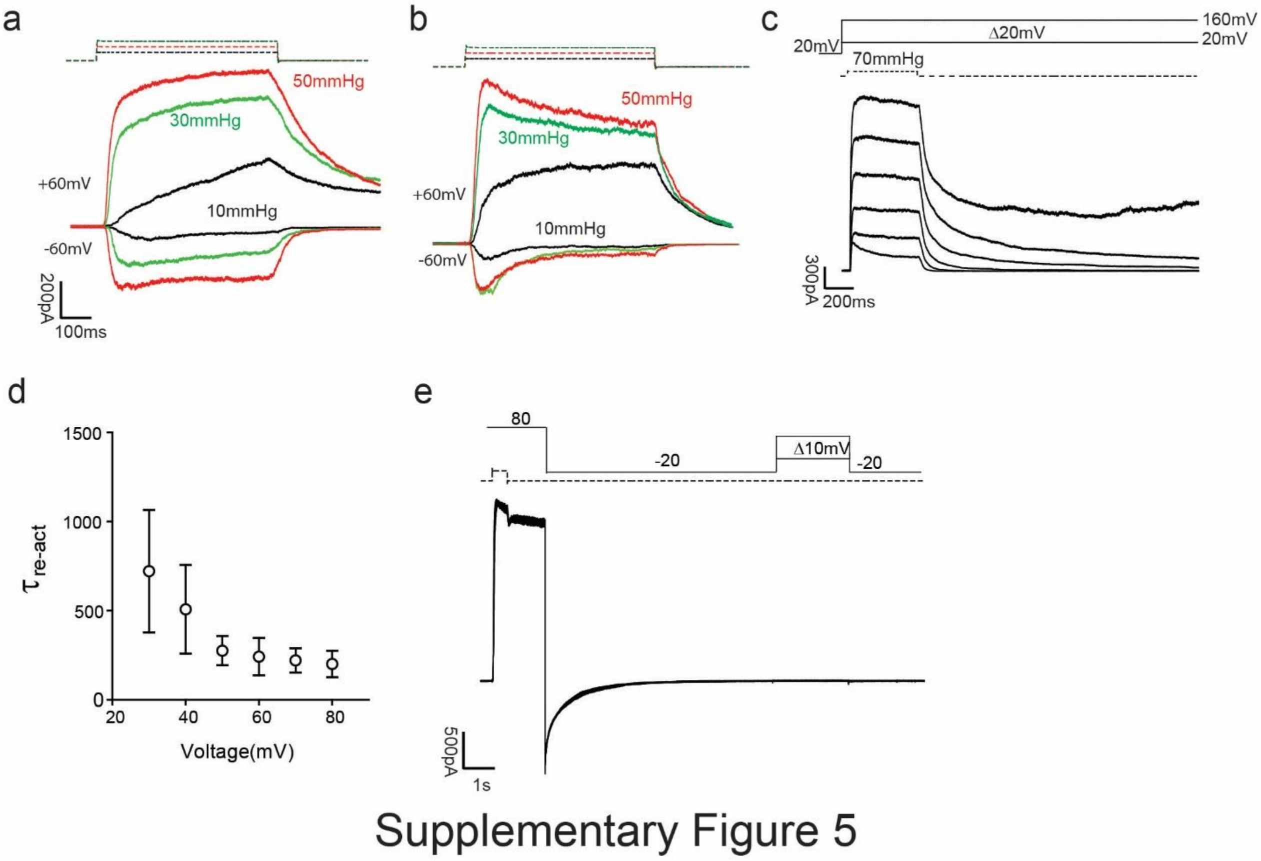

Supplementary Figure 5. Pressure activation of wt PIEZO1 in presence of Yoda1. (Related to Fig. 6)

Increasing pressure stimuli were applied to outside-out patches pulled from N2A cells overexpressing wt PIEZO1 at a constant voltage of either -60mV (inward currents) and +60mV (outward currents). a) Yoda1 (5µM) in the intracellular solution b) Vehicle in the intracellular solution. Note the difference in kinetics in presence of Yoda1. c) The wild type PIEZO1 was subject to a family of pressure stimulations at increasing voltages (from 60 to 160mV) and the deactivation phase was extended to 2 seconds. In less than 5% of the cells and at voltages higher than 140mV it was possible to observe a reactivation phase as for the R2482H/K mutant. d) Reactivation time constants referring to Fig. 6a. Note how the time course of re-activation accelerates at more depolarized voltages. e) R2482K was subject to a similar protocol as in Fig. 6b. A saturating pressure pulse at 80mV was applied to force the channel to reactivate and switch to voltage-gated mode. Following a 2 s deactivation period at -20mV (to induce the inactivation gate to close) a family of 1 s steps at increasing voltages (in absence of pressure) was

applied. PIEZO1 R2482K cannot be activated by the sole application of voltage when the deactivation occurs at negative voltages. This event forces the inactivation gate to close and the channel into an inactive state.

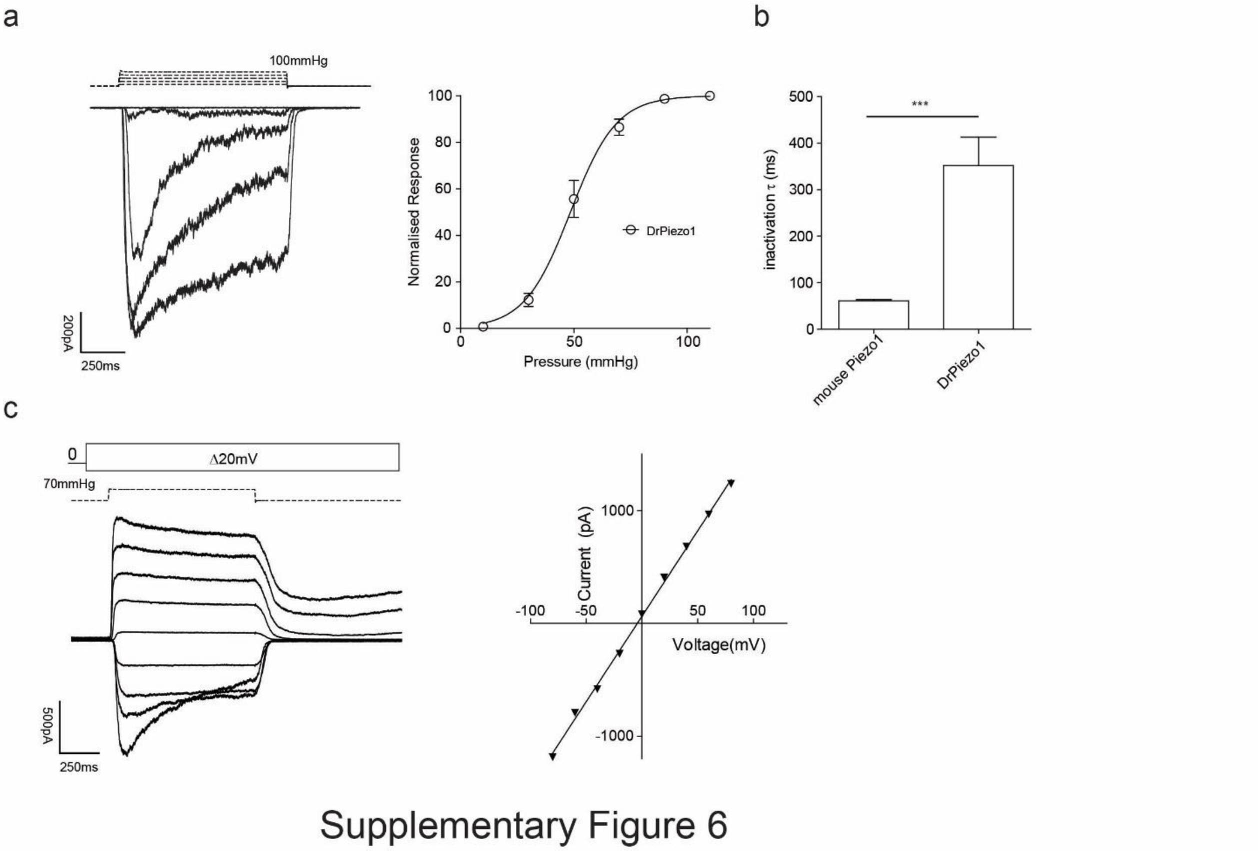

Supplementary Figure 6. Pressure sensitivity and I/V relationship of the DrPIEZO1 (zebrafish) (Related to Figure 7)

a) Patches pulled from N2a *Piezo1 -/-* cell line overexpressing the ZPIEZO1 were clamped at -60mV and subject to increasing pressure (10 to 110mmHg). Fitting of the pressure response curve gave a P_{50} value of 51 ± 2.7 (n=9) mmHg. b) Exponential fitting of the time course of inactivation showed a significantly slower kinetic for ZPIEZO1 in comparison to mPIEZO1 (Student's *t*-test *P* = 0.0003). c) I/V relationship for ZPIEZO1. Saturating pressure pulses were applied to patches at voltages ranging from -100mv to +80mV. Peak currents were plotted against voltage and showed a linear I/V relationship.

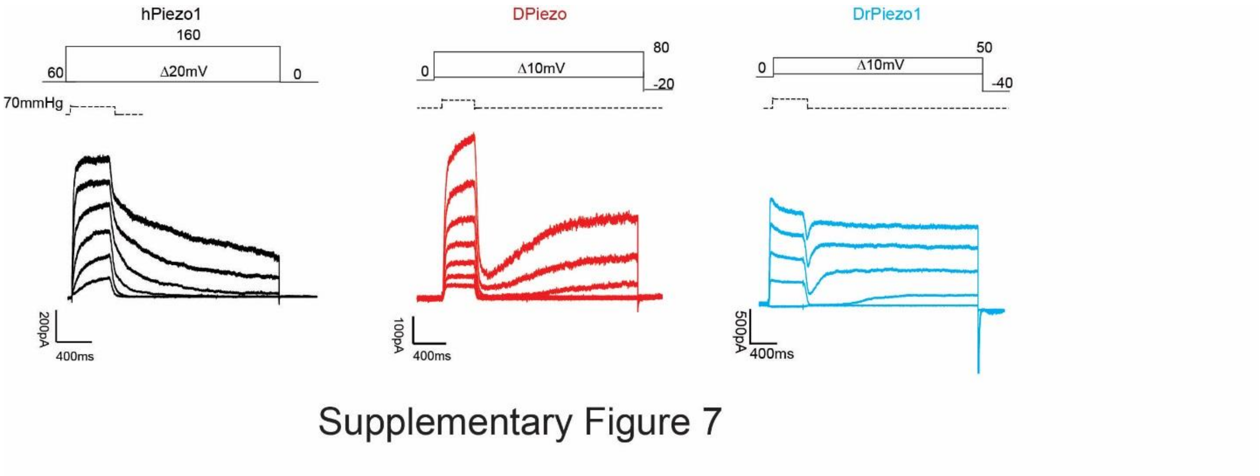

Supplementary Figure 7. Voltage gating of vertebrates and invertebrates PIEZO1 orthologues (Related to Fig. 7)

The hPIEZO1, DPIEZO and DrPIEZO1 were subject to a family of pressure stimulations at increasing voltages (as indicated) and the deactivation phase was extended to 2 seconds. The human PIEZO1 deactivated more slowly at depolarized voltages. After an initial deactivation the DPIEZO and DrPIEZO1, at voltages above 40mV and 20mV respectively, exhibited a sag followed by a reactivation phase.

Supplementary Table 1. (Related to Fig. 4)

Inactivation time course for Xerocytosis mutants. Currents were elicited by applying a saturating pressure pulse (500ms) at -60mV. P values are reported in comparison to wt (ANOVA with Dunnett's Post-hoc test).

	Inactivation tau (-60mV)	n	*p*
Piezo1 wt	60.5 ± 7.8	8	
Piezo1 R2482H	319.5 ± 55.4	13	<0.0001
Piezo1 R1353P	83.8 ± 2.17	6	0.043
Piezo1 A2036T	88.3 ± 5.6	5	0.025
Piezo1 T2143M	120.6 ± 10.9	5	0.001

Supplementary Table 2. (Related to Fig. 6)

Estimation of gating charge movements in pressure-gated and voltage-gated modes for PIEZO1 R2482H/K mutants and wild type channels.

	e_0 Pressure-Gated mode	n	e_0 Voltage-Gated mode	n
Piezo1 R2482K	2.2 ± 0.4	6	6.0 ± 1.1	10
Piezo1 R2482H	2.4 ± 0.4	19	6.3 ± 1.5	8
Piezo1 wt (Yoda5μM)	/		5.3 ± 1.7	5

Reported values within each column are not significantly different. Pressure-gated mode Student's t test (P=0.82). Voltage-gated mode ANOVA (Dunnett's post test P=0.84, 92,65 respectively for R2482K Vs 2482H, R2482K Vs Piezo1 wt Yoda, R2482H Vs Piezo1 wt Yoda).

Supplementary Table 3. List of primers for cloning and mutagenesis. Capitalized letters are used for cloning purposes.

Primer Name	Sequence
DrPiezo1-for	AACTGCACCTCGGTTCTGCCACC atggagcttcaggtggtatgcggattg
DrPiezo1-rev	CCTGCAGAAGCTTAATTCA tcagttgtgattcttctctctcgtccacttgatc
R2482K-for	ggtggttggcaagtttgtgaagggcttcttcagcgagatc
R2482K-rev	gatctcgctgaagaagcccttcacaaacttgccaaccacc
R2482H-for	ggtggttggcaagtttgtgcacggcttcttcagcgagatc
R2482H-for	gatctcgctgaagaagccgtgcacaaacttgccaaccacc
R1353P-for	gacagatgaagcgcatccctgccaaacaggagaagtacag
R1353P-rev	ctgtacttctcctgtttggcagggatgcgcttcatctgtc
A2036T-for	ggcaccatggtcatcgaccgtaccctctacctgcgcaagactg
A2036T-rev	cagtcttgcgcaggtagagggtacggtcgatgaccatggtgcc
T2143M-for	ccgtcatggactgggtgtggatggacaccacgctgtccctgtcc
T2143M-rev	ggacagggacagcgtggtgtccatccacacccagtccatgacgg
